# Reading the mind in the eyes and cognitive ability in schizophrenia- and autism spectrum disorders

**DOI:** 10.1017/S0033291723002052

**Published:** 2023-12

**Authors:** Rebecca Alvarez, Eva Velthorst, Amy Pinkham, Kelsey A. Ludwig, Jorge Alamansa, Sebastian B. Gaigg, David L. Penn, Philip D. Harvey, Anne-Kathrin Fett

**Affiliations:** 1 Department of Psychology, City, University of London, London, UK; 2 Community Mental Health Department GGZ Noord-Holland-Noord, Heerhugowaard, the Netherlands; 3 Department of Psychology, The University of Texas at Dallas, Richardson, TX, USA; 4 Department of Psychiatry, University of North Carolina, Chapel Hill, NC, USA; 5 Department of Psychology and Neuroscience, University of North Carolina, Chapel Hill, NC, USA; 6 School of Behavioural and Health Sciences, Australian Catholic University, Melbourne, Victoria, Australia; 7 University of Miami Miller School of Medicine, Miami, FL, USA; 8 Research Service, Bruce W. Carter Medical Center, Miami, FL, USA; 9 Department of Psychosis Studies, Institute of Psychiatry, Psychology and Neuroscience (IoPPN), King's College London, London, UK

**Keywords:** Autism spectrum disorder, cognitive ability, reading the mind in the eyes, schizophrenia, social cognition

## Abstract

**Background:**

Schizophrenia (SZ) and autism spectrum disorders (ASD) are characterized by difficulties in theory of mind (ToM). We examined group differences in performance on a ToM-related test and associations with an estimated IQ.

**Methods:**

Participants [*N* = 1227, SZ (*n* = 563), ASD (*n* = 159), and controls (*n* = 505), 32.2% female] completed the Reading the Mind in the Eyes Test (RMET) and assessments of cognitive ability. Associations between IQ and group on RMET were investigated with regression analyses.

**Results:**

SZ (*d* = 0.73, *p* < 0.001) and ASD (*d* = 0.37, *p* < 0.001) performed significantly worse on the RMET than controls. SZ performed significantly worse than ASD (*d* = 0.32, *p* = 0.002). Adding IQ to the model, SZ (*d* = 0.60, *p* < 0.001) and ASD (*d* = 0.44, *p* < 0.001) continued to perform significantly worse than controls, but no longer differed from each other (*d* = 0.13, *p* = 0.30). Small significant negative correlations between symptom severity and RMET performance were found in SZ (PANSS positive: *r* = −0.10, negative: *r* = −0.11, both *p* < 0.05). A small non-significant negative correlation was found for Autism Diagnostic Observation Schedule scores and RMET in ASD (*r* = −0.08, *p* = 0.34).

**Conclusions:**

SZ and ASD are characterized by impairments in RMET. IQ contributed significantly to RMET performance and accounted for group differences in RMET between SZ and ASD. This suggests that non-social cognitive ability needs to be included in comparative studies of the two disorders.

## Background

Social cognition is a multifaceted set of cognitive processes that include the ability to perceive, understand, and interpret social and emotional information based on different social cues from the environment (Sasson, Pinkham, Carpenter, & Belger, 2011) that are necessary to guide behavior within social interactions (Baron-Cohen, Jolliffe, Mortimore, & Robertson, 1997). Theory of mind (ToM), or mental state attribution, is a specific social cognitive function that refers to the ability to infer others' mental states, intentions, thoughts, and emotions (Fernandes, Cajão, Lopes, Jerónimo, & Barahona-Corrêa, 2018; Kuo & Eack, 2020). It has been found to be a source of difficulty in a variety of psychiatric disorders that are also characterized by difficulties in social functioning (Pinkham, 2019; Velikonja, Fett, & Velthorst, 2019). ToM is most impaired in individuals with schizophrenia spectrum disorders (SZ) and in those with autism spectrum disorders (ASD) (Baron-Cohen et al., 1997; Pennington & Ozonoff, 1996; Sasson et al., 2011; Velikonja et al., 2019). Importantly, meta-analyses suggest that ToM difficulties might be a key contributing factor to problems in social functioning (Fett et al., 2011; Halverson et al., 2019).

A growing number of studies have directly compared social cognitive performance in SZ and ASD and contrasted their performance to that of healthy controls (Fernandes et al., 2018; Hajdúk, Pinkham, Penn, Harvey, & Sasson, 2022; Morrison et al., 2019; Oliver et al., 2021). The largest study to date included 101 individuals with ASD, 92 IQ matched individuals with SZ, and 101 controls. In this study, ASD and SZ performed similarly on tasks measuring ToM, emotion recognition, social perception, and attributional style (Pinkham et al., 2020). Findings were in line with previous work which also found that adults with SZ and ASD performed significantly worse on ToM-related tasks than controls, with the SZ and ASD group performing similarly (Craig, Hatton, Craig, & Bentall, 2004). In contrast, earlier, smaller studies reported that individuals with SZ present with more difficulties than individuals with ASD in identifying others' beliefs and evaluating social and emotional content (Pinkham et al., 2012; Sasson et al., 2011). The discrepancy in findings might be explained by methodological differences between studies, including the cognitive tests employed, socio demographic differences between the samples, and overall small sample sizes. A meta-analysis of 14 studies that included 857 individuals showed no overall difference between ASD and SZ in Reading the Mind in the Eyes Test (RMET) performance, a task which is thought to measure aspects of ToM, however, differences between studies were statistically significantly heterogeneous (Oliver et al., 2021).

Importantly, SZ and ASD are characterized by difficulties in both social and non-social cognition (Heinrichs & Zakzanis, 1998; Velikonja et al., 2019). Degrees of overlap between performance on the RMET and the intelligence quotient (IQ) in SZ groups when compared to healthy control groups suggest that general cognitive processes might partly account for poor social cognitive performance, at least on some ToM-related tasks (Pinkham, Penn, Green, & Harvey, 2016). The RMET requires the correct identification of complex emotional states of others from a photograph of a facial expression that is presented along with four words that describe different emotional states. Thus, the participant needs to pay attention, retrieve previous emotional experiences and associated information and needs to read and know the words that are presented. It is therefore likely that particularly good verbal ability (Peterson & Miller, 2012), verbal memory (Dalkner et al., 2019) and attention enhance RMET test performance (Kynast et al., 2020). Additionally, the RMET relies on the detection of subtle facial clues (Oakley, Brewer, Bird, & Catmur, 2016), and RMET performance may therefore be facilitated by subtle cognitive processes, such as attention to detail and (analogic) reasoning [i.e. the ability to identify shared similarities in different situations, (Seo et al., 2020)].

Factor analytic studies show that social and non-social cognitive domains are only partially dissociated (van Hooren et al., 2008), emphasizing the importance of considering the role of cognitive ability when comparing individuals with SZ and ASD with each other and with controls. Systematic differences in non-social cognitive functions may impact performance on social cognitive tests. For example, a lack of attention, or verbal or memory problems may compromise social cognitive performance, while strong non-social cognitive skills may enable individuals to compensate for social cognitive difficulties (Eack et al., 2013; Velikonja et al., 2019). A recent study found that adjusting for non-social cognitive performance eliminated differences in performance between schizophrenia and healthy controls participants on the RMET, but not five other social cognition measures (Meissner & Brigham, 2001). To summarize, while the growing body of research shows that ToM is a source of significant difficulty in both ASD and SZ, differences in the degree of such difficulties, and the possible role of non-social cognitive processes in this context, remain less well studied. This multi-center study uses the largest sample of 563 individuals with SZ, 159 individuals with ASD and 505 controls to date, to directly compare SZ and ASD groups on RMET performance, and to explore the impact of non-social cognition. Based on previous research, we hypothesized that (1) the SZ and ASD groups would perform comparably to each other on the REMT and that both groups would perform significantly worse than controls, and that (2) IQ and symptom severity would account for some of the social cognitive difficulties in ASD and SZ.

## Method

### Participants

The total study sample consisted of 563 participants with a diagnosis of a SZ, 159 participants with an ASD, and 505 control participants. Data were derived from four different studies (subsamples), including 86 participants from a social functioning study at the Amsterdam University Medical Center [AUMC, SWIPE study; (Pos et al., 2019)], 116 participants from autism research projects at City, University of London (CUoL), 102 participants of the *Decision Making and Context Processing in Psychosis* (DECOP) (for details see e.g. (Hanssen, Krabbendam, Robberegt, & Fett, 2020; Hanssen, van Buuren, Van Atteveldt, Lemmers-Jansen, & Fett, 2022) study conducted at King's College London (KCL), and 923 participants of the *Social Cognition Psychometric Evaluation* study [SCOPE; for details see (Ludwig, Pinkham, Harvey, Kelsven, & Penn, 2017; Pinkham, Harvey, & Penn, 2018)]. Autistic individuals across sites were diagnosed by qualified clinicians or clinical teams within local health services according to the Diagnostic and Statistical Manual [DSM-IV-TR or DSM-5; (APA, 2000; 2013)] or International Statistical Classification of Diseases version 10 [ICD; (WHO, 2019)] criteria. In addition, research teams collected further corroborating information through the Autism Diagnostic Observation Schedule (Lord, Luyster, Gotham, & W., 2012). The AUMC SZ participants were diagnosed by their treating clinicians according to DSM-IV-TR (APA, 2000). Participants at the UK sites had ICD based diagnoses (WHO, 2019). SCOPE participants were diagnosed with SZ via the Mini International Neuropsychiatric Interview (MINI), and the SCID Psychosis Module according to DSM (First, Spitzer, & Gibbon, 2002). Recruitment procedures for control participants varied by site. At CUoL controls were recruited partly through an existing database of individuals who had taken part in previous studies and who had indicated that they were happy to be contacted again, and partly through advertisement flyers in the local area, word of mouth and e-mail circulation. Control participants were only enrolled if they confirmed that they had no personal or family history of psychiatric disorders, drug or alcohol abuse. At KCL controls were recruited online via websites (e.g. Gumtree, Callforparticipants), and recruitment circular emails at the Institute of Psychiatry, Psychology and Neuroscience. Controls were precluded from participation for meeting criteria for any diagnosed psychiatric disorder, or if they had a first-degree family member with a history of psychosis. The UTD led SCOPE study recruited age/gender matched control participants through community flyers and online advertisements. Controls were precluded from participation for meeting criteria for any Axis I/II disorder according to the DSM-IV, or if they had a first-degree family member with a history of psychosis. All projects had local ethical approval.

### Measures and materials

#### Reading the Mind in the Eyes Test

The RMET is an assessment of social cognition in the domain of ToM (Baron-Cohen et al., 1997). Research shows that the RMET has acceptable psychometric properties in individuals with SZ (Pinkham et al., 2016). Similarly, the SCOPE study concluded that the RMET has adequate psychometric properties for studying social cognition in adults with ASD, relative to healthy individuals (Morrison et al., 2019). All sites used the 36-item version of the RMET, during which participants were instructed to identify the mental state through pictures of the eye region of the face. Participants had to choose one of four words which best described the individuals' thoughts or feelings (e.g. serious, alarmed, relaxed, amused, ashamed etc.). The RMET total score was calculated as the sum of the correct responses out of 36. The proportion of correct responses (score of 0 to 36, divided by the total possible score) was included in the analyses. Sites either used the paper and pencil or an electronic version (see Procedures section). The proportions of correct RMET scores and SDs by study group and study subsample are shown in Tables 1 and 2, respectively.

**Table 1.** Sample characteristics and task performance by study group

Variable	Overall sample	Control group	Schizophrenia group	Autism spectrum disorder group	Group differences (*p* < 0.05)
Participants (*N*, (%))	1227 (100)	505 (41.16)	563 (45.88)	159 (12.96)	
Gender (female, %)	32.2	34.9	28.4	13.21	C = SZ > ASD
	M (s.d.)	M (s.d.)	M (s.d.)	M (s.d.)	
Age (years)	36.89 (13.38)	38.02 (13.75)	37.22 (12.59)	32.18 (13.98)	C = SZ > ASD
Proportion RMET correct	0.65 (0.15)	0.70 (0.12)	0.60 (0.15)	0.64 (0.17)	C > ASD > SZ
IQ	99.96 (14.87)	103.10 (13.14)	95.10 (14.83)	107.24 (14.69)	ASD > C > SZ

Abbreviations. ASD, autism spectrum disorder group; IQ, estimated intelligence quotient; C, control group; RMET, Reading the Mind in the Eyes Test; SZ, schizophrenia group.

**Table 2.** Sample characteristics and task performance by subsample and group

	Control group	Schizophrenia group	Autism spectrum disorder group	Total N (1227)
***Subsample***				
**AUMC**				
Participants (N, %)		86		86
Female (%)		15.10		
Race (not white %)		–		
	M (s.d.)	M (s.d.)	M (s.d.)	
Age (years)		24.84 (4.10)		
Proportion RMET correct		0.66 (0.13)		
WAIS 4 subtests		90.68 (14.21)		
PANSS pos		12.2 (5.0)		
PANSS neg		15.6 (5.0)		
PANSS gen		27.3 (6.8)		
**CUoL**				
Participants (*N*)	60		56	116
Female (%)	28.30		17.90	
Race (not white %)	–		–	
	M (s.d.)	M (s.d.)	M (s.d.)	
Age (years)	48.55 (13.96)		46.71 (12.59)	
Proportion RMET correct	0.73 (0.11)		0.66 (0.13)	
WAIS FSIQ	109.24 (14.83)		110.27 (17.48)	
ADOS			8.63 (3.37)	
**KCL**				
Participants (N)	52	50		102
Female (%)	30.08	18.0		
Race (not white %)	38.46	74.00		
	M (s.d.)	M (s.d.)	M (s.d.)	
Age (years)	34.27 (9.54)	36.01 (9.89)		
Proportion RMET correct	0.75 (0.12)	0.57 (0.18)		
WASI 2-subtest IQ	111.56 (11.62)	98.44 (13.08)		
PANSS pos		13.32 (4.38)		
PANSS neg		15.06 (5.10)		
PANSS gen		28.55 (5.60)		
**SCOPE**				
Participants (N)	393	427	103	923
Female (%)	36.40	32.30	37.42	
Race (not white %)	45.29	49.41	11.65	
	M (s.d.)	M (s.d.)	M (s.d.)	
Age (years)	36.96 (13.51)	39.86 (12.51)	24.28 (6.17)	
Proportion RMET correct	0.69 (0.12)	0.59 (0.15)	0.65 (0.19)	
WRAT-3	101.15 (12.38)	95.55 (14.98)	105.64 (12.81)	
PANSS pos		16.36 (2.98)		
PANSS neg		14.29 (5.31)		
PANSS gen		31.14 (7.85)		
ADOS			10.88 (3.09)	

Abbreviations. ADOS, autism diagnostic observation schedule; AUMC, Amsterdam Medical Centre; CUoL, City Univeristy of London; KCL, King's College London; PANSS, Positive and Negative Symptom Scale (gen, general; neg, negative; pos, positive); RMET, Reading the Mind in the Eyes Test; SCOPE, Social Cognition Psychometric Evaluation Study; WAIS, Wechsler Adult Intelligence Scale; WRAT, Wide range achievement test.

#### Estimated IQ

At the AUMC, IQ estimates were determined by an abbreviated four subtest version of the Wechsler Adult Intelligence Scale (WAIS), including digit symbol-coding, information, block design, and arithmetic. This version has been found to accurately assess general intellectual ability in both clinical and research settings (Velthorst et al., 2013; Wechsler, Coalson, & Raiford, 2008). CUoL estimated IQ using the WAIS-IV^UK^ (Wechsler et al., 2008), in which subtests for a verbal comprehension index (vocabulary, similarities, information), working memory index (arithmetic, digit span), perceptual reasoning index (block design, matrix reasoning, visual puzzles) and processing speed index (digit symbol coding and symbol search) are combined to derive a measure of full-scale IQ. The KCL DECOP study utilized the Wechsler Abbreviated Scale of Intelligence (WASI) two subtest version (Wechsler, 2011), which includes the vocabulary and matrix reasoning subtests to estimate IQ. The SCOPE study utilized the Wide Range Achievement Test-3 Reading subscale (WRAT) to estimate IQ scores (Wilkinson & Robertson, 2017). All IQ tests were scaled to an average of 100 with a standard deviation of 15.

#### Autism diagnostic observation schedule (ADOS)

The ADOS is an activity-based, semi-structured interview assessment to evaluate social interaction, play, imaginative use of materials, and communication skills in individuals suspected to have (or already diagnosed with) ASD. The assessment is conducted through one of five 30–60-min observational schedules, chosen depending on an individuals' age and verbal ability. The ADOS module 4 (adults and adolescents) has four domain scores: (1) communication, (2) social interaction, (3) communication–social interaction total and (4) stereotyped behaviors and restricted interests. Higher scores on the domains indicate more severe difficulties. Here we report total ADOS scores, as indicator of the overall ASD symptom severity (Lord et al., 2012). The ADOS was administered and scored at the CUoL and SCOPE sites by individuals trained to research reliability on this instrument.

#### Positive and negative syndrome scale

The positive and negative syndrome scale (PANSS) is a semi-structured 30 item clinical interview that assesses the severity of symptoms of SZ and general psychopathology (Kay, Fiszbein, & Opler, 1987). Consisting of three subscales, the PANSS distinguishes between positive, negative, and general symptoms, rated on a seven-point Likert scale with ratings of 3 and higher indicating clinically relevant symptoms. The PANSS was completed by participants with SZ as assessment of symptom severity.

#### Procedures

*Amsterdam University Medical Center*. Data came from participants of the SWIPE study, which included help-seeking individuals (age 18–35) who were referred to the clinic or day-treatment of the Department of Early Psychosis, Amsterdam and experienced their first psychotic episode <4 years prior to their first assessment. All participants completed informed consent prior to study participation. The study comprised two approximately 2-h long sessions to determine the level of symptoms (including with the PANSS), cognitive and social functioning, including the Dutch version of the RMET. The investigation was carried out in accordance with the latest version of the Declaration of Helsinki. The study was approved by the Medical Ethical Committee of the AUMC, Amsterdam.

*City University of London*. Data came from several research projects at this site. Recruitment for the different projects was supported through ongoing advertisement of research opportunities through relevant local organizations, social media, and word of mouth. All projects assessed relevant clinical diagnoses, which were ascertained through review of available clinical records and direct assessments of cognition (WAIS), and reciprocal social communication and autistic traits [ADOS, Social Responsiveness Scale (SRS)]. Participants completed the RMET either online or on A4 sheets of laminated paper with four-word choice options of emotion words and a question (Baron-Cohen et al., 1997). All participants signed consent forms as part of the individual projects they participated in, which were all approved by the Psychology Department Research Ethics Committee of City University.

*King's College London*. Participants were recruited via the South London and Maudsley National Health Service, Oxleas NHS, Northeast London Foundation Trust and South Essex Partnership University NHS Foundation Trust, the SLaM ‘Consent for Consent c4c’ initiative, and with support of the Mental Health Research Network. All participants gave informed consent prior to participating in the study. The study comprised two visits that included completion of several tasks and questionnaires [e.g., see (Hanssen et al., 2020; Hanssen et al., 2022)]. Participants first completed the RMET on a computer, which included all steps of the paper version and completed the WASI vocabulary and matrix reasoning subtests during the first testing session. During the second session, participants completed the PANSS. The study was approved by the London-Harrow Research Ethics Committee [14/LO/0071].

*SCOPE*. The SCOPE study was conducted at multiple sites including UTD, the University of Miami Miller School of Medicine, Southern Methodist University, and the University of North Carolina at Chapel Hill [e.g., see (Pinkham et al., 2016, 2018, 2020)]. SZ were recruited from local mental health clinics in the Dallas, Chapel Hill, and Miami metropolitan areas, and autistic individuals were recruited from the UTD Autism Research Collaborative. Control participants were recruited via advertisements in the local communities of each site. Once assessed by inclusion criteria, participants were asked to complete informed consent. The computerized RMET standard version was administered, along with the WRAT-3 reading subscale (Wilkinson & Robertson, 2017). Additionally, symptom severity was measured in SZ using the PANSS (Kay et al., 1987). ASD diagnoses were confirmed using the autism diagnostic observation schedule [ADOS-2; (Lord et al., 2012)]. The study was approved by the IRBs at all research sites.

### Data analysis

Data analysis was conducted using the IBM Statistical Package for the Social Sciences (SPSS version 26). SPSS general linear model (GLM) analyses were carried out to examine group differences in RMET performance between controls, SZ, and ASD. The dependent variable of this analysis was the proportion of correct RMET answers, with group (C, SZ, ASD) as categorical predictor variable. We first investigated group differences in RMET. In the second model, we included IQ. A third model included race as additional predictor to control for race biases. All GLM analyses were controlled for age and sex. As the inclusion of diagnosis groups varied systematically by site, site could not be added to the model. However, we conducted a separate sensitivity analysis to confirm the overall results in in the SCOPE sample which included all three participant groups. Significant group effects were further analyzed using the pairwise comparisons. We report the mean difference in the proportion correct RMET answers, as well as the Cohen's d. Finally, Pearson Correlation analyses were conducted to investigate the relationship between symptoms, IQ and RMET performance within each diagnostic group. Additional exploratory analyses on the associations with education are reported in the online Supplementary Material.

## Results

### Sample description

Descriptive statistics for the overall sample and analyses to explore group differences can be found in Table 1. The overall sample mean age was 36.89 years (s.d. = 13.38), with 32.2% of the 1227 participants identifying as female. The groups differed significantly in age (*p* < 0.0001), gender composition (*p* < 0.0001) and IQ (*p* < 0.0001). Specifically, the SZ and control group included significantly more females and were significantly older than the ASD group. ASD had the highest average estimated IQ, followed by controls and SZ, with the lowest average estimated IQ. Sample characteristics by study site are shown in Table 2.

### Group differences in RMET performance

The groups differed significantly in RMET performance (Table 3a). Pairwise comparisons showed that the SZ (*M_d_* = 0.10; s.e. = 0.009, *p* < 0.001, *d* = 0.73) and the ASD group (*M_d_* = 0.06; s.e. = 0.13, *p* < 0.001, *d* = 0.34) performed significantly worse than controls. The SZ group performed significantly worse than the ASD group (*M_d_* = 0.04; s.e. = 0.13; *p* < 0.002, *d* = 0.32).

**Table 3.** General linear models

			SS	df	*F*	*p* value
**a) Model 1: RMET and group**						
*Independent variable*^a^						
Group			2.63	2	70.46	< 0.001
*Estimated marginal means*^a^	RMET PC	s.e.				
Controls	0.70	0.01				
ASD	0.66	0.01				
SZ	0.60	0.01				
				
**b) Model 2: RMET, Group and IQ**						
*Independent variable*^a^			SS	df	*F*	*p* value
Group			1.08	2	34.34	< 0.001
IQ			5.51	1	400.16	< 0.001
*Estimated marginal means*^a^	RMET PC	s.e.				
Controls	0.69	0.01				
ASD	0.63	0.01				
SZ	0.63	0.01				
						
**c) Model 3: RMET, Group, IQ and race**						
*Independent variable*^b^			SS	df	*F*	*p* value
Group			1.19	2	38.61	< 0.001
IQ			4.73	1	325.26	< 0.001
Race			0.07	1	7.31	0.01
*Estimated marginal means*^b^	RMET PC	s.e.				
Controls	0.68	0.01				
ASD	0.62	0.01				
SZ	0.62	0.01				

Abbreviations. ASD, autism spectrum disorder group; IQ, estimated intelligence quotient; PC, proportion correct; RMET, Reading the Mind in the Eyes Test; s.e., standard error; SZ, schizophrenia group.

*Note*. ^a^Age and gender are controlled for in the analysis. ^b^Age, gender, and race (White v. Non-White) are controlled for in the analysis.

### The role of IQ in RMET performance

**Figure 1.**
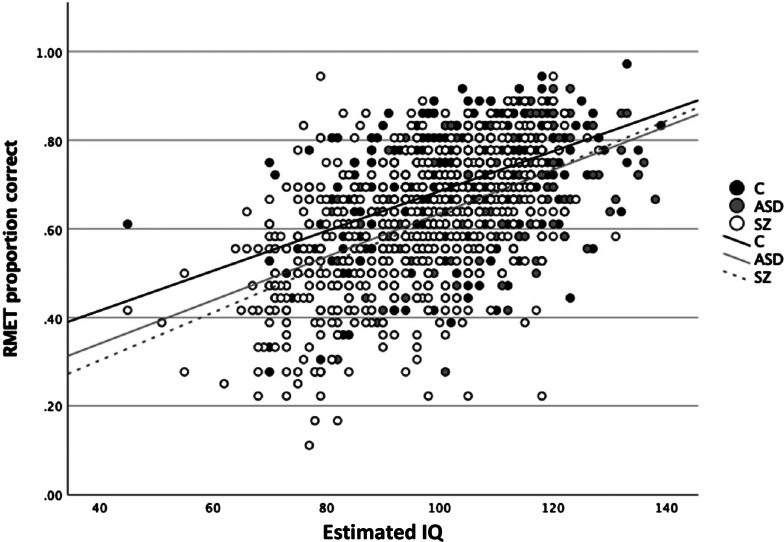
Scatterplot of RMET proportion correct against estimated IQ by group. *Note*. C, control group; ASD, autism spectrum disorder group; SZ, schizophrenia group.

IQ was a significant predictor of RMET performance (Table 3b). The group effect on RMET remained significant, with both SZ (*M_d_* = 0.07; s.e. = 0.08; *p* < 0.001, *d* = 0.60) and ASD (*M_d_* = 0.06; s.e. = 0.12; *p* < 0.001, *d* = 0.44) groups continuing to perform worse than controls. However, the SZ group no longer differed significantly from the ASD group on RMET performance (*M_d_* = 0.01; s.e. = 0.12, *p* = 0.30, *d* = 0.13), showing that group differences in IQ between ASD and SZ account for group differences on the RMET. In all participant groups IQ and RMET performance were strongly and significantly correlated (ASD: *r* = 0.47, *p* < 0.001; SZ: *r* = 0.53, *p* < 0.001; C: *r* = 0.48, *p* < 0.001, see Fig. 1). An interaction term between group and IQ was included in model b to test whether the relationship between IQ and RMET differed between groups. The interaction term was not significant [*F*_(1,1203)_ = 1.58, *p* = 0.21]. Finally, race was added to the analyses to determine whether any group differences were confounded by the unequal sample composition (Table 3c). While race was a significant predictor of RMET performance, whereby white individuals performed better than non-white individuals (*M_d_* = 0.02; s.e. = 0.15, *p* = 0.03, *d* = 0.52), the effects of group and IQ remained significant.

### Sensitivity analysis in the SCOPE sample

The sensitivity analysis confirmed the overall results. The first analyses showed significant group differences on the RMET [*F*_(2, 918)_ = 45.82, *p* < 0.001], whereby controls showed better performance than ASD and SZ, and ASD showed better performance than SZ, and all three groups differed significantly from each other at *p* < 0.01. When IQ was added to the model the overall group effect remained significant [*F*_(1, 918)_ = 31.56, *p* < 0.001], however while the difference between controls and ASD and SZ remained significant at *p* < 0.001, ASD and SZ no longer differed from each other significantly (*p* = 0.39).

### RMET performance and symptom severity

A correlation analysis was performed to assess the relationship between PANSS symptom severity and RMET performance in SZ. Small, but significant negative associations were found between PANSS positive (*r* = −0.10, *p* < 0.05) and PANSS negative (*r* = −0.11, *p* < 0.01) symptoms and RMET performance, showing that higher symptom levels were associated with lower RMET scores. There was no significant correlation between the PANSS general subscale and RMET performance (*r* = −0.01, *p* = 0.81). The correlation between the ADOS score and RMET performance in the AD group was not significant (*r* = −0.08, *p* = 0.34). The associations remained significant when IQ was controlled for in the analysis, albeit somewhat weaker for negative symptoms. The correlations between symptoms RMET and IQ are shown in online Supplementary Table S3.

## Discussion

This study investigated RMET performance in the largest sample of individuals with a diagnosis of SZ and ASD and controls to date. We examined whether estimated non-social cognitive ability (IQ) accounted for observed group differences in RMET performance and explored associations between symptoms and RMET performance in individuals with SZ and ASD. The results of this study yielded three key findings. First, results support previous research indicating difficulties in ToM-related mental state attribution in both SZ and ASD groups compared to controls (Fernandes et al., 2018; Pinkham et al., 2016; Sasson et al., 2007, 2011). Second, IQ performance was significantly associated with RMET performance in all groups (*r*'s = 0.47 to 0.53). Third, in the unadjusted analyses SZ performed worse on the RMET than ASD, however when adjusted for IQ, our results were in line with a large recent meta-analysis of 13 studies that found no differences in RMET performance between individuals with ASD and SZ (Oliver et al., 2021).

Our finding highlights the importance of including estimates for IQ in comparative studies of social cognition, specifically the RMET. Previous studies have yielded mixed findings with respect to the associations between RMET and IQ. Some meta-analytic work in ASD suggested that RMET performance is unrelated to full-scale IQ estimates of non-social cognitive performance and negatively correlated with performance IQ. The author cautions, however, that the included samples were mostly very small and that the results might be confounded. Further these studies included individuals had a very broad age range (6–60 years), which may dilute effects within specific age groups (Peñuelas-Calvo, Sareen, Sevilla-Llewellyn-Jones, & Fernández-Berrocal, 2019). However, moderate to strong associations have previously been reported for controls (Peñuelas-Calvo et al., 2019) and samples including controls, ASD and SZ (Baker, Peterson, Pulos, & Kirkland, 2014; Peñuelas-Calvo, Sareen, Porras-Segovia, Cegla-Schvatzman, & Fernandez-Berrocal, 2021; Seo et al., 2020). Dodell-Feder, Ressler, and Germine (2020) further suggested that the RMET may be particularly influenced by education, culture, and other factors that are associated with global estimates of cognitive performance. We conducted an exploratory analysis to assess what role, if any, education had on RMET performance in our subsamples. Education was significantly correlated with RMET performance at only one of the three sites with available data (online Supplementary Table S1**)**. However, when IQ and education were simultaneously included in the regression models to predict RMET, education no longer predicted RMET performance significantly (online Supplementary Table S2). Finally, as ASD individuals with better cognitive functioning may be more inclined to participate in research, investigators need to take special care to recruit representative samples to examine whether our findings generalize to individuals with ASD who experience cognitive difficulties.

We found small negative correlations between the two symptom-related measures used in SZ and ASD, and RMET performance. The associations between the PANSS positive and negative scale and RMET performance in the SZ group (*r* = −0.10 and −0.11, respectively) were small but statistically significant. The association between the ADOS and RMET performance in the ASD group had a comparable small effect size but was not significant (*r* = −0.08), likely due to the smaller sample size. Overall, the findings support previous work that suggests that symptoms and social cognition are relatively independent in both ASD and SZ, as previously reported by others (Barendse, Hendriks, Thoonen, Aldenkamp, & Kessels, 2018; Dominguez, Viechtbauer, Simons, van Os, & Krabbendam, 2009; Fett & Maat, 2013; Oakley et al., 2016), albeit not consistently (Del Valle Rubido et al., 2018). There are different explanations for this. It is possible that both phenomena might be related to different disorder-related neural processes. For example, psychotic symptoms have been associated with aberrant dopaminergic neurotransmission and functioning in mid-brain areas, whereas (social) cognitive performance problems have been related to frontal and parietal regions. Alternatively, it might be that assessment of real-world social functioning through the ADOS and PANSS negative scale that require the utilization of multiple social cognitive skills in complex context, are not predicted well by the highly structured social cognitive RMET assessment in lab settings, which probes only a relatively narrow facet of social cognition. Further research is necessary to elucidate this question.

Our results suggest that, regardless of diagnoses, training non-social cognitive domains in addition to social cognitive ones may be useful to maximize social cognitive improvement, and possibly to yield a larger impact on patients' overall functioning and quality of personal relationships (Fett et al., 2011; Halverson et al., 2019). This suggestion is consistent with results of combined social-cognitive and neurocognitive training in people with particularly chronic and severe schizophrenia (Lindenmayer et al., 2018). However, such training needs to consider the levels of non-social cognitive ability of the participant, to ensure that pace, level of explanation and aid for attention and memory are adjusted to the right level for optimal learning.

The findings of the current study need to be interpreted in the light of several limitations. First, there was some cross-subsample variation in our assessments of IQ, and not every subsample included all diagnostic groups, potential confounding differences could not be accounted for. Even though the WAIS and its abbreviated forms were used to assess cognitive ability in the AUMC, CUoL and KCL samples, different subtests were used. However, for the SZ and ASD groups there were no differences in IQ between the SCOPE and KCL or AUMC samples, respectively. While controls in the CUoL and KCL samples did not differ in terms of IQ, both did differ significantly from controls in the SCOPE sample. This could be due to differences between the WAIS and the WRAT-3 estimates of cognitive ability. However, previous research indicates that the WRAT and WAIS-R have similar relationships to levels of general intelligence scores (Spruill & Beck, 1986). Similarly, the WAIS short forms showed consistency in measuring general intelligence scores when compared to the longer WAIS-R form (Pilgrim, Meyers, Bayless, & Whetstone, 1999; van Ool et al., 2018). Moreover, for controls, RMET performance was also lower in the SCOPE sample compared to the CUoL and KCL samples. Together this suggests that the SCOPE control sample was generally lower performing in terms of cognitive ability, rather than group differences that were caused by the nature of the utilized cognitive measures. Second, the RMET is exclusively showing images of faces of White individuals, and it is possible that there is a bias towards better recognizing emotional expressions in own-race faces (Meissner & Brigham, 2001). We therefore conducted additional exploratory analyses in the samples with available data on race (SCOPE and KCL), to investigate whether any group differences between SZ, ASD and controls were accounted for by differences in sample composition of White and non-White participants in the respective groups. Race was significantly correlated with RMET performance (partial correlation controlling for group status: *r* = −0.22), suggesting a race bias in the RMET whereby White individuals performed better than non-White individuals. The current findings highlight the need for culturally sensitive versions of the task and the importance of reporting race and/or ethnicity effects (Nagendra et al., 2018, 2022). However, when race was controlled for in the GLM analyses group effects between SZ, ASD and controls remained significant (Table 3, model c). Finally, it is important to note that our findings may not generalize to other tests of ToM. While the RMET taps into ToM, it also relies on emotion recognition abilities. Not all ToM tests have an emotion recognition component. Moreover, the RMET might be more strongly correlated to cognitive ability compared to other social cognitive tasks, because many of the options for mind states in the RMET are of higher reading levels than most social cognitive tests (Eddy & Hansen, 2020). To address this, future research may utilize a battery of social cognitive tasks in conjunction with the RMET to further assess the relationship between IQ and social cognition. Finally, to disentangle which non-social cognitive processes may particularly interact with RMET performance, a battery including different non-social cognitive tasks is recommended.

In conclusion, this large study of RMET performance in ASD and SZ supports previous findings that both groups perform worse on the RMET compared to controls. It also indicates that the ASD and SZ group performed similarly when non-social cognitive ability was considered in the statistical analysis. Together these results suggest the necessity to carefully consider and assess non-social cognitive ability in future comparative studies of social cognitive ability.

## Supporting information

Alvarez et al. supplementary material Alvarez et al. supplementary material

## Supplementary material

The supplementary material for this article can be found at https://doi.org/10.1017/S0033291723002052

## References

[ref1] APA. (2000). Diagnostic and statistical manual of mental disorders: DSM-IV-TR (4th / text revision. ed.). Washington, DC: American Psychiatric Association.

[ref2] APA. (2013). Diagnostic and statistical manual of mental disorders: DSM-5 (5th ed.). London, Washington, DC: American Psychiatric Association.

[ref3] Baker, C. A., Peterson, E., Pulos, S., & Kirkland, R. A. (2014). Eyes and IQ: A meta-analysis of the relationship between intelligence and “Reading the Mind in the Eyes”. Intelligence, 44, 78–92. doi: 10.1016/j.intell.2014.03.001

[ref4] Barendse, E. M., Hendriks, M. P. H., Thoonen, G., Aldenkamp, A. P., & Kessels, R. P. C. (2018). Social behaviour and social cognition in high-functioning adolescents with autism spectrum disorder (ASD): Two sides of the same coin? Cognitive Processing, 19(4), 545–555. doi: 10.1007/s10339-018-0866-5 29959562

[ref5] Baron-Cohen, S., Jolliffe, T., Mortimore, C., & Robertson, M. (1997). Another advanced test of theory of mind: Evidence from very high functioning adults with autism or Asperger syndrome. Journal of Child psychology and Psychiatry, 38(7), 813–822. doi: 10.1111/j.1469-7610.1997.tb01599.x 9363580

[ref6] Craig, J. S., Hatton, C., Craig, F. B., & Bentall, R. P. (2004). Persecutory beliefs, attributions and theory of mind: Comparison of patients with paranoid delusions, Asperger's syndrome and healthy controls. Schizophrenia Research, 69(1), 29–33. doi: 10.1016/s0920-9964(03)00154-3 15145468

[ref7] Dalkner, N., Bengesser, S. A., Birner, A., Fellendorf, F. T., Hamm, C., Platzer, M., … Reininghaus, E. Z. (2019). The relationship between “Eyes Reading” ability and verbal memory in bipolar disorder. Psychiatry Research, 273, 42–51. doi: 10.1016/j.psychres.2019.01.015 30639563

[ref8] Del Valle Rubido, M., McCracken, J. T., Hollander, E., Shic, F., Noeldeke, J., Boak, L., … Umbricht, D. (2018). In search of biomarkers for Autism Spectrum Disorder. Autism Research, 11(11), 1567–1579. doi: 10.1002/aur.2026 30324656 PMC6282609

[ref9] Dodell-Feder, D., Ressler, K. J., & Germine, L. T. (2020). Social cognition or social class and culture? On the interpretation of differences in social cognitive performance. Psychological Medicine, 50(1), 133–145. doi: 10.1017/s003329171800404x 30616706

[ref10] Dominguez, M. G., Viechtbauer, W., Simons, C. J., van Os, J., & Krabbendam, L. (2009). Are psychotic psychopathology and neurocognition orthogonal? A systematic review of their associations. Psychological Bulletin, 135(1), 157–171. doi: 10.1037/a0014415 19210058

[ref11] Eack, S. M., Bahorik, A. L., McKnight, S. A. F., Hogarty, S. S., Greenwald, D. P., Newhill, C. E., … Minshew, N. J. (2013). Commonalities in social and non-social cognitive impairments in adults with autism spectrum disorder and schizophrenia. Schizophrenia Research, 148(1), 24–28. doi: 10.1016/j.schres.2013.05.013 23768814 PMC3732579

[ref12] Eddy, C. M., & Hansen, P. C. (2020). Predictors of performance on the reading the mind in the eyes test. PLOS ONE, 15(7), e0235529. doi: 10.1371/journal.pone.0235529 32701998 PMC7377373

[ref13] Fernandes, J. M., Cajão, R., Lopes, R., Jerónimo, R., & Barahona-Corrêa, J. B. (2018). Social cognition in schizophrenia and autism spectrum disorders: A systematic review and meta-analysis of direct comparisons. Frontiers in Psychiatry, 9, 504. doi: 10.3389/fpsyt.2018.00504 30459645 PMC6232921

[ref14] Fett, A. K., & Maat, A. (2013). Social cognitive impairments and psychotic symptoms: What is the nature of their association? Schizophrenia Bulletin, 39(1), 77–85. doi: 10.1093/schbul/sbr058 21697150 PMC3523914

[ref15] Fett, A. K., Viechtbauer, W., Dominguez, M. D., Penn, D. L., van Os, J., & Krabbendam, L. (2011). The relationship between neurocognition and social cognition with functional outcomes in schizophrenia: A meta-analysis. Neuroscience and Biobehavioral Reviews, 35(3), 573–588. doi: 10.1016/j.neubiorev.2010.07.001 20620163

[ref16] First, M., Spitzer, R. L., & Gibbon M. W. J. (2002). Structured Clinical Interview for DSM-IV-TR Axis I Disorders, Research Version, Patient Edition (SCID-I/P) New York: John Wiley & Sons.

[ref17] Hajdúk, M., Pinkham, A. E., Penn, D. L., Harvey, P. D., & Sasson, N. J. (2022). Heterogeneity of social cognitive performance in autism and schizophrenia. Autism Research, 15(8), 1522–1534. doi: 10.1002/aur.2730 35460541

[ref18] Halverson, T. F., Orleans-Pobee, M., Merritt, C., Sheeran, P., Fett, A. K., & Penn, D. L. (2019). Pathways to functional outcomes in schizophrenia spectrum disorders: Meta-analysis of social cognitive and neurocognitive predictors. Neuroscience and Biobehavioral Reviews, 105, 212–219. doi: 10.1016/j.neubiorev.2019.07.020 31415864

[ref19] Hanssen, E., Krabbendam, L., Robberegt, S., & Fett, A.-K. (2020). Social and non-social reward learning reduced and related to a familial vulnerability in schizophrenia spectrum disorders. Schizophrenia Research, 215, 256–262. doi: 10.1016/j.schres.2019.10.019 31753593

[ref20] Hanssen, E., van Buuren, M., Van Atteveldt, N., Lemmers-Jansen, I. L., & Fett, A.-K. J. (2022). Neural, behavioural and real-life correlates of social context sensitivity and social reward learning during interpersonal interactions in the schizophrenia spectrum. Australian & New Zealand Journal of Psychiatry, 56(1), 59–70. doi: 10.1177/00048674211010327 34006142 PMC8721616

[ref21] Heinrichs, R. W., & Zakzanis, K. K. (1998). Neurocognitive deficit in schizophrenia: A quantitative review of the evidence. Neuropsychology, 12(3), 426–445. doi: 10.1037/0894-4105.12.3.426 9673998

[ref22] Kay, S. R., Fiszbein, A., & Opler, L. A. (1987). The positive and negative syndrome scale (PANSS) for schizophrenia. Schizophrenia Bulletin, 13(2), 261–276. doi: 10.1093/schbul/13.2.261 3616518

[ref23] Kuo, S. S., & Eack, S. M. (2020). Meta-analysis of cognitive performance in neurodevelopmental disorders during adulthood: Comparisons between autism spectrum disorder and schizophrenia on the Wechsler adult intelligence scales. Frontiers in psychiatry, 11, 187. doi: 10.3389/fpsyt.2020.00187 32273855 PMC7114889

[ref24] Kynast, J., Quinque, E. M., Polyakova, M., Luck, T., Riedel-Heller, S. G., Baron-Cohen, S., … Schroeter, M. L. (2020). Mindreading from the eyes declines with aging – evidence from 1603 subjects. Frontiers Aging Neuroscience, 12, 550416. doi: 10.3389/fnagi.2020.550416 PMC7656776 33192452

[ref25] Lindenmayer, J.-P., Khan, A., McGurk, S. R., Kulsa, M. K. C., Ljuri, I., Ozog, V., … Thanju, A. (2018). Does social cognition training augment response to computer-assisted cognitive remediation for schizophrenia? Schizophrenia Research, 201, 180–186. 29910120 10.1016/j.schres.2018.06.012

[ref26] Lord, C., Luyster, R., Gotham, K., & W., G. (2012). Autism diagnostic observation schedule (2nd ed.). Torrence, CA: Western Psychological Services.

[ref27] Ludwig, K. A., Pinkham, A. E., Harvey, P. D., Kelsven, S., & Penn, D. L. (2017). Social cognition psychometric evaluation (SCOPE) in people with early psychosis: A preliminary study. Schizophrenia Research, 190, 136–143. doi: 10.1016/j.schres.2017.03.001 28302395 PMC5735418

[ref28] Meissner, C. A., & Brigham, J. C. (2001). Thirty years of investigating the own-race bias in memory for faces: A meta-analytic review. Psychology, Public Policy, and Law, 7(1), 3.

[ref29] Morrison, K. E., Pinkham, A. E., Kelsven, S., Ludwig, K., Penn, D. L., & Sasson, N. J. (2019). Psychometric evaluation of social cognitive measures for adults with autism. Autism Research, 12(5), 766–778. doi: 10.1002/aur.2084 30770676 PMC6499650

[ref30] Nagendra, A., Orleans-Pobee, M., Spahnn, R., Monette, M., Sosoo, E. E., Pinkham, A. E., & Penn, D. L. (2022). How often do US-based schizophrenia papers published in high-impact psychiatric journals report on race and ethnicity?: A 20–year update of Lewine and Caudle (1999). Journal of Mental Health, 31(5), 649–656. doi: 10.1080/09638237.2020.1837356 33166190

[ref31] Nagendra, A., Twery, B. L., Neblett, E. W., Mustafic, H., Jones, T. S., Gatewood, D., & Penn, D. L. (2018). Social cognition and African American men: The roles of perceived discrimination and experimenter race on task performance. Psychiatry Research, 259, 21–26. doi: 10.1016/j.psychres.2017.09.074 29024856

[ref32] Oakley, B. F. M., Brewer, R., Bird, G., & Catmur, C. (2016). Theory of mind is not theory of emotion: A cautionary note on the reading the mind in the eyes test. Journal of Abnormal Psychology, 125(6), 818–823. doi: 10.1037/abn0000182 27505409 PMC4976760

[ref33] Oliver, L. D., Moxon-Emre, I., Lai, M.-C., Grennan, L., Voineskos, A. N., & Ameis, S. H. (2021). Social cognitive performance in schizophrenia spectrum disorders compared with autism spectrum disorder: A systematic review, meta-analysis, and meta-regression. JAMA Psychiatry, 78(3), 281–292. doi: 10.1001/jamapsychiatry.2020.3908 33291141 PMC7724568

[ref34] Pennington, B. F., & Ozonoff, S. (1996). Executive functions and developmental psychopathology. Journal of Child Psychology and Psychiatry, 37(1), 51–87. 8655658 10.1111/j.1469-7610.1996.tb01380.x

[ref35] Peñuelas-Calvo, I., Sareen, A., Porras-Segovia, A., Cegla-Schvatzman, F.-B., & Fernandez-Berrocal, P. (2021). The association between reading the mind in the eyes test performance and intelligence quotient in children and adolescents with Asperger syndrome. Frontiers in Psychiatry, 12, 642799. doi: 10.3389/fpsyt.2021.642799 33854452 PMC8039142

[ref36] Peñuelas-Calvo, I., Sareen, A., Sevilla-Llewellyn-Jones, J., & Fernández-Berrocal, P. (2019). The “reading the mind in the eyes” test in autism-spectrum disorders comparison with healthy controls: A systematic review and meta-analysis. Journal of Autism and Developmental Disorders, 49(3), 1048–1061. doi: 10.1007/s10803-018-3814-4 30406435

[ref37] Peterson, E., & Miller, S. (2012). The eyes test as a measure of individual differences: How much of the variance reflects verbal IQ? Frontiers in Psychology, 3. doi: 10.3389/fpsyg.2012.00220 PMC3389807 22783217

[ref38] Pilgrim, B. M., Meyers, J. E., Bayless, J., & Whetstone, M. M. (1999). Validity of the Ward Seven-Subtest WAIS-III short form in a neuropsychological population. Applied Neuropsychology, 6(4), 243–246. doi: 10.1207/s15324826an0604_7 10635439

[ref39] Pinkham, A. E. (2019). Metacognition in psychosis. Journal of Experimental Psychopathology, 10(2), 204380871984114. doi: 10.1177/2043808719841146

[ref40] Pinkham, A. E., Harvey, P. D., & Penn, D. L. (2018). Social cognition psychometric evaluation: Results of the final validation study. Schizophrenia Bulletin, 44(4), 737–748. doi: 10.1093/schbul/sbx117 28981848 PMC6007629

[ref41] Pinkham, A. E., Morrison, K. E., Penn, D. L., Harvey, P. D., Kelsven, S., Ludwig, K., & Sasson, N. J. (2020). Comprehensive comparison of social cognitive performance in autism spectrum disorder and schizophrenia. Psychological Medicine, 50(15), 2557–2565. doi: 10.1017/s0033291719002708 31576783

[ref42] Pinkham, A. E., Penn, D. L., Green, M. F., & Harvey, P. D. (2016). Social cognition psychometric evaluation: Results of the initial psychometric study. Schizophrenia Bulletin, 42(2), 494–504. doi: 10.1093/schbul/sbv056 25943125 PMC4753585

[ref43] Pinkham, A. E., Sasson, N. J., Beaton, D., Abdi, H., Kohler, C. G., & Penn, D. L. (2012). Qualitatively distinct factors contribute to elevated rates of paranoia in autism and schizophrenia. Journal of Abnormal Psychology, 121(3), 767–777. doi: 10.1037/a0028510 22686868

[ref44] Pos, K., Franke, N., Smit, F., Wijnen, B. F. M., Staring, A. B. P., Van der Gaag, M., … Schirmbeck, F. (2019). Cognitive behavioral therapy for social activation in recent-onset psychosis: Randomized controlled trial. Journal of Consulting and Clinical Psychology, 87(2), 151–160. doi: 10.1037/ccp0000362 30570309

[ref45] Sasson, N. J., Pinkham, A. E., Carpenter, K. L. H., & Belger, A. (2011). The benefit of directly comparing autism and schizophrenia for revealing mechanisms of social cognitive impairment. Journal of Neurodevelopmental Disorders, 3(2), 87–100. doi: 10.1007/s11689-010-9068-x 21484194 PMC3188289

[ref46] Sasson, N. J., Tsuchiya, N., Hurley, R., Couture, S. M., Penn, D. L., Adolphs, R., & Piven, J. (2007). Orienting to social stimuli differentiates social cognitive impairment in autism and schizophrenia. Neuropsychologia, 45(11), 2580–2588. doi: 10.1016/j.neuropsychologia.2007.03.009 17459428 PMC2128257

[ref47] Seo, E., Koo, S. J., Kim, Y. J., Min, J. E., Park, H. Y., Bang, M., … An, S. K. (2020). Reading the mind in the eyes test: Relationship with neurocognition and facial emotion recognition in non-clinical youths. Psychiatry Investigation, 17(8), 835–839. doi: 10.30773/pi.2019.0281 32791819 PMC7449835

[ref48] Spruill, J., & Beck, B. (1986). Relationship between the WAIS-R and Wide Range Achievement Test-Revised. Educational and Psychological Measurement, 46(4), 1037–1040. doi: 10.1177/001316448604600424

[ref49] van Hooren, S., Versmissen, D., Janssen, I., Myin-Germeys, I., à Campo, J., Mengelers, R., … Krabbendam, L. (2008). Social cognition and neurocognition as independent domains in psychosis. Schizophrenia Research, 103(1), 257–265. doi: 10.1016/j.schres.2008.02.022 18434094

[ref50] van Ool, J. S., Hurks, P. P. M., Snoeijen-Schouwenaars, F. M., Tan, I. Y., Schelhaas, H. J., Klinkenberg, S., … Hendriksen, J. G. M. (2018). Accuracy of WISC-III and WAIS-IV short forms in patients with neurological disorders. Developmental Neurorehabilitation, 21(2), 101–107. doi: 10.1080/17518423.2016.1277799 28152329

[ref51] Velikonja, T., Fett, A. K., & Velthorst, E. (2019). Patterns of nonsocial and social cognitive functioning in adults with autism spectrum disorder: A systematic review and meta-analysis. JAMA Psychiatry, 76(2), 135–151. doi: 10.1001/jamapsychiatry.2018.3645 30601878 PMC6439743

[ref52] Velthorst, E., Levine, S. Z., Henquet, C., de Haan, L., van Os, J., Myin-Germeys, I., & Reichenberg, A. (2013). To cut a short test even shorter: Reliability and validity of a brief assessment of intellectual ability in schizophrenia – a control-case family study. Cognitive Neuropsychiatry, 18(6), 574–593. doi: 10.1080/13546805.2012.731390 23167265

[ref53] Wechsler, D. (2011). Wechsler abbreviated scale of intelligence *(*WASI-II*)* (2nd ed.). San Antonio, TX: The Psychological Corporation.

[ref54] Wechsler, D., Coalson, D. L., & Raiford, S. E. (2008). WAIS-IV technical and interpretive manual. San Antonio, TX: Pearson.

[ref55] WHO. (2019). ICD-10 : international statistical classification of diseases and related health problems : tenth revision. (9241546492 (v.1), 9241546530 (v.2), 9241546549 (v.3)). Geneva: World Health Organization. Retrieved from https://apps.who.int/iris/handle/10665/42980.

[ref56] Wilkinson, G. S., & Robertson, G. J. (2017). Wide Range Achievement Test professional manual (5th edn). Bloomington, MN: NCS Pearson, Inc.

