# Supplementary material for: Reading the mind in the eyes and cognitive ability in schizophrenia- and autism spectrum disorders
Source: Psychol Med. 2023 Jul 31;53(16):7913–22. doi: 10.1017/S0033291723002052 (PMC10755246; doi:10.1017/S0033291723002052)
Supplement: Alvarez et al. supplementary material [file S0033291723002052sup001.docx]

**SUPPLEMENTARY MATERIAL**

Reading the Mind in the Eyes and cognitive ability in Schizophrenia- and Autism Spectrum Disorders

**Supplementary Table 1.** Correlations between gender, age, education, IQ and RMET by subsample

| **Subsample** | | **Variable** |  | **RMET** | **Female**  **gender** | **Age** | **Education** |
| --- | --- | --- | --- | --- | --- | --- | --- |
| **AUMC** |  | Female gender | | 0.01 |  |  |  |
|  |  | Age | | 0.02 | -0.02 |  |  |
|  |  | Education (years) | | -0.05 | 0.20 | 0.06 |  |
|  |  | IQ | | **0.42*** | -0.07 | -0.17 | 0.03 |
| **CUoL** |  | Female gender | | 0.10 |  |  |  |
|  |  | Age | | -0.08 | 0.19* |  |  |
|  |  | Education (not assessed) | | - | - | - |  |
|  |  | IQ | | **0.40**** | 0.05 | 0.02 |  |
| **KCL** |  | Female gender | | 0.15 |  |  |  |
|  |  | Age | | -0.10 | -0.12 |  |  |
|  |  | Education (level) | | 0.17 | **0.20*** | -0.10 |  |
|  |  | IQ | | **0.54**** | **0.21*** | 0.12 | **0.26*** |
| **SCOPE** |  | Gender | | **-0.05*** |  |  |  |
|  |  | Age | | **-0.20**** | **0.16**** |  |  |
|  |  | Education (years) | | **0.34**** | -0.03 | **-0.10**** |  |
|  |  | IQ | | **0.57**** | **-0.16**** | **-0.29**** | **0.34**** |

** Correlation is significant at the 0.01 level (2-tailed). * Correlation is significant at the 0.05 level (2-tailed). Significant correlations are in bold. Abbreviations. AUMC = Amsterdam Medical Centre, CUoL = City Univeristy of London, IQ = estimated Intelligence Quotient, KCL = King’s College London, RMET = Reading the Mind in the Eyes Test, SCOPE = Social Cognition Psychometric Evaluation Study.

**Supplementary Table 2. Regression analysis of education and IQ on RMET scores by subsample**

| **Subsample** | **Predictor variable*** | **Beta** | **t** | **p-value** |
| --- | --- | --- | --- | --- |
| AUMC | Education (years) | -0.19 | -1.6 | 0.11 |
|  | IQ | 0.46 | 4.02 | <0.001 |
| KCL | Education (level) | -0.01 | -0.08 | 0.93 |
|  | IQ | 0.58 | 7.49 | <0.001 |
| SCOPE | Education (years) | 0.09 | 2.93 | 0.003 |
|  | IQ | 0.53 | 17.00 | <0.001 |

Note. *Age and gender are controlled for in the analysis.

Abbreviations. AUMC = Amsterdam Medical Centre, IQ = estimated Intelligence Quotient, KCL = King’s College London, SCOPE = Social Cognition Psychometric Evaluation Study.

**Supplementary Table 3. Correlations between RMET, IQ and symptoms**

| **Variable** | **PANSS P6** | **PANSS pos** | **PANSS neg** | **PANSS gen** | **ADOS** |
| --- | --- | --- | --- | --- | --- |
| IQ | -0.09 | 0.009 | -0.09* | 0.06 | -0.13 |
| RMET | -0.06 | -0.10* | -0.11** | -0.01 | -0.08 |
| RMET corrected for IQ | -0.03 | -0.11** | -0.07* | -0.04 | -0.02 |

Note. One-tailed test significant at **p < 0.01, * p < 0.05. P6 suspiciousness/persecution, pos = positive, neg = negative, gen = general.
